# Population Genetic Structure of Codling Moth, *Cydia pomonella* (L.) (Lepidoptera: Tortricidae), in Different Localities and Host Plants in Chile

**DOI:** 10.3390/insects11050285

**Published:** 2020-05-06

**Authors:** Alejandra Basoalto, Claudio C. Ramírez, Blas Lavandero, Luis Devotto, Tomislav Curkovic, Pierre Franck, Eduardo Fuentes-Contreras

**Affiliations:** 1 Center in Molecular and Functional Ecology, Facultad de Ciencias Agrarias, Universidad de Talca, Casilla 747, Talca, Chile; abasoalto@utalca.cl; 2 Center in Molecular and Functional Ecology, Instituto de Ciencias Biológicas, Universidad de Talca, Casilla 747, Talca, Chile; clramirez@utalca.cl; 3 Laboratorio de Control Biológico, Instituto de Ciencias Biológicas, Universidad de Talca, Casilla 747, Talca, Chile; blavandero@utalca.cl; 4 Instituto de Investigaciones Agropecuarias, Centro Regional de Investigación Quilamapu, Casilla 426, Chillán, Chile; ldevotto@inia.cl; 5 Facultad de Ciencias Agronómicas, Universidad de Chile, Casilla 1004, Santiago, Chile; tcurkovi@uchile.cl; 6 UR1115 Plantes et Systèmes de Culture Horticoles, INRAe, 228 Route de l’Aérodrome CS 40509, Domaine Saint Paul, Site Agroparc, CEDEX 09, 84914 Avignon, France; pierre.franck@inra.fr

**Keywords:** Bayesian assignment, genetic structure, microsatellites, TESS

## Abstract

The codling moth, *Cydia pomonella* (L.) (Lepidoptera: Tortricidae), is a major pest introduced to almost all main pome fruit production regions worldwide. This species was detected in Chile during the last decade of the 19th century, and now has a widespread distribution in all major apple-growing regions. We performed an analysis of the genetic variability and structure of codling moth populations in Chile using five microsatellite markers. We sampled the codling moth along the main distribution area in Chile on all its main host-plant species. Low genetic differentiation among the population samples (*F*_ST_ = 0.03) was found, with only slight isolation by distance. According to a Bayesian assignment test (TESS), a group of localities in the coastal mountain range from the Bío-Bío Region formed a distinct genetic cluster. Our results also suggest that the codling moth that invaded the southernmost locality (Aysén Region) had two origins from central Chile and another unknown source. We did not find significant genetic differentiation between codling moth samples from different host-plant species. Our results indicate high genetic exchange among codling moth populations between the different Chilean regions and host plants.

## 1. Introduction

The codling moth, *Cydia* *pomonella* (L.) (Lepidoptera: Tortricidae), is an invasive species with a widespread distribution in temperate areas with pome fruit cultivation worldwide [1,2]. Originated in central Eurasia, it is currently the main insect pest in most temperate pome fruit production regions of Europe, Northeast China, South Africa, Australia, New Zealand, North America, and the southern cone of South America [3].

Study of the codling moth population genetic structure has received increasing attention in the last few decades [4], because this knowledge can be useful for improving the design of eradications or integrated area-wide pest management strategies using the sterile insect technique (SIT), host plant removal, or mating disruption [5,6,7,8]. For instance, the population’s genetic structure might inform the effective distance over which codling moth individuals disperse and reproduce, and therefore determine the spatial scales at which pest management must be applied [9,10,11]. At larger spatial scales, the genetic study of the codling moth might reveal genetic diversity centers and invasion routes [12] and help to improve the monitoring and quarantine actions to prevent the codling moth establishment in new areas [1,8,13].

The spatial patterns of genetic diversity among codling moth populations may reflect its relatively recent global distribution [11], which was most likely the result of founder effects following genetic bottlenecks caused mainly by human-mediated dispersal through pome fruit cultivation [4,13]. Long distance transportation of infested material can produce this passive dispersal of the codling moth, and explain the relatively high gene flow detected between geographically distant locations [4,14,15,16].

The codling moth is an oligophagous species that develops within the fruit of a few species of cultivated Rosaceae, such as apples (*Malus domestica* Borkdhausen), pears (*Pyrus communis* L.), quinces (*Cydonia oblonga* Mill.), and less frequently, apricots (*Prunus armeniaca* L.) and plums (*Prunus domestica* L.) [17]. In addition, it uses the walnut (*Juglans regia* L.) as a host plant in the Juglandaceae [17]. Singular codling moth host-plant races have been described on walnuts in California [18,19] and on apricots in Armenia [20] Genetic differentiation between codling moth populations on apple, walnut, and apricot host-plant strains have been reported in Switzerland and South Africa [10,21].

Dominant molecular markers, such as AFLP and random amplified polymorphic DNA (RAPD), have found significant differentiation between populations of codling moth at the regional and local scales in South Africa [22], central Europe (Italy and Germany) [23,24], Iran [25], and Pakistan [26]. Co-dominant markers, such as microsatellites, have been developed for the codling moth [27,28], and used to evaluate the structure of local populations from France [4,9,16], Switzerland [10], Croatia [29], Greece [15,30], China [8,11], and Chile [14,31,32] with variable results. Low *F*_ST_ values [33] and little or no isolation by distance at the country level were observed in these latter studies, although higher population differentiation was detected in Switzerland [10], and in China too [8,11]. Topographic barriers between different pome fruit production areas, differences in pest management measures (e.g., use of organophosphate insecticides and/or mating disruption), and other anthropogenic influences on the codling moth dispersal could explain such differences in genetic structure [13]. For instance, population differences due to management (e.g., insecticide resistant strains) might explain differences in thermal requirements in some states of the US [34] or developmental times and the diapause propensity in France [35], both affecting phenology and pest management. Furthermore, the frequency of insecticide sprays seems to also influence the population structure at local scales [4,9,15,16,29].

The populations of the codling moth from the main apple producing regions in central Chile exhibit a low genetic structure [14,31], but with significant isolation by distance (IBD), and detectable levels of geographical clustering [14]. In Chile, the codling moth has a wide latitudinal distribution (approximately 30°–47° S). The southernmost localities in Aysén Region were invaded during the first decade of the 2000 [36]. In addition, codling moth populations in Chile develop on apple, pear, quince, and walnut host plants [37,38]. In rural areas of the main apple production regions, back-yard fruit trees have been widespread since colonial times [38,39]. These trees are usually maintained without insecticide treatments, and are important sources of codling moths, impacting the meta-population dynamics [32,40].

No information is available on the genetic structure of the codling moth populations among the main apple production regions, or among populations using different host plants in Chile. We expect that isolated codling moth populations from southern Chile, where no pome fruit industry exists, would show higher genetic differentiation than the populations from central Chile, where apple production for exportation is concentrated. Therefore, the main objective of this research was to characterize the population-level genetic structures of codling moth populations in Chile, all along their distribution area, which covers a latitudinal range of nearly 1400 km. Samples from all host-plant species of the codling moth in Chile were included (apples, pears, quinces, and walnuts), with the aim of evaluating whether the development of population differentiation between them was present.

## 2. Materials and Methods

### 2.1. Insect Material

**Figure 1:**
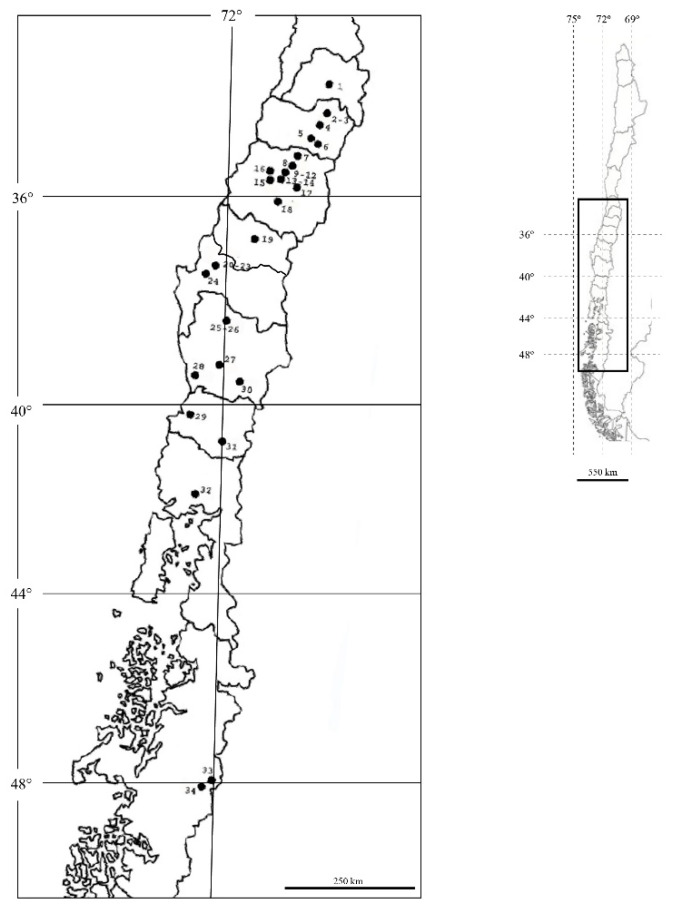
Map of Chile indicating localities where the codling moth samples were collected. Number corresponds to each location detailed in Table 1. Map indicates degrees of latitude (south) and longitude (west).

Diapausing larvae were collected using cardboard traps wrapped around tree trunks in 2008 and 2009 autumns; there were one to five individual trees per population sample. A total of 34 population samples were collected from 26 locations along a 1400 km transect in Chile (Table 1, Figure 1). In addition, three samples from a single location with the most common host plants in France (apples, pears, and walnuts) were included for comparative purposes as an outgroup [14]. Samples from the main host plants for the codling moth in Chile [38] were included: apples, pears, quinces, and walnuts (21, 4, 3, and 6 population samples, respectively). No samples were obtained from plums or apricots, because codling moth infestations on these stone fruits are very uncommon in Chile [38]. Samples from apple trees were collected in more locations than not, because apples are the most common and widespread host plant in Chile. When available, population samples from the different host-plant species were collected at the same location (e.g., at Panguilemo and Santa Juana locations). Cardboard traps were brought to the laboratory, and the larvae were preserved in Eppendorf tubes with ethanol (96%) for microsatellite analysis. The outgroup samples corresponded to codling moth diapausing larvae collected in autumn 2003 in three distinct apple, pear and apple orchards at the INRA Gotheron research station, France (orchards 6, 7 and 8 in [9]).

### 2.2. Microsatellite Analysis

DNA templates were extracted from diapausing larvae according to the salting out procedure [41]. An abdominal body section of each larva (about 1 mm width) was dissected and homogenized with pestle inside plastic tubes with 300 µL TNES buffer (Tris-HCl 50 mM, pH 7.5, NaCl 400 mM, EDTA 20 mM, SDS 0.5%), and incubated overnight with proteinase K (10 mg/mL) at 37 °C for Chilean and 55 °C for French samples. Then, DNA was precipitated with NaCl 5 M, followed by centrifugation at 10,000 rpm. Finally, DNA extracts were washed twice with 350 μL cold ethanol, dried, and suspended in ultrapure distilled water to get a final concentration of about 10 ng/µL. Five microsatellite loci, *Cp1.60*, *Cp1.62*, *Cp2.129*, *Cp5.24,* and *Cp6.46* [9,27], were examined in a total of 609 larvae from Chile and 109 larvae from France. This set of five microsatellites was selected because of their level of polymorphism, ability to discriminate codling moth populations at large international geographical scales, and their very low frequency of null alleles [16,30]. Furthermore, these primers were compatible to be used in a multiplex PCR reaction. PCR reactions were performed in 12 µL reaction volumes containing 10 mM Tris–HCl, pH 9, 50 mM KCl, 200 µM each dNTP, 0.4 µM each primer, 1.5 mM MgCl_2_, one unit of Taq DNA polymerase, and 0.1 mg/mL BSA with 2 µL of DNA template. The forward primer for each pair was labeled with 5′-carboxyfluorescein (FAM) or 6-carboxy-1,4-dichloro-2′,4′,5′,7′-tetra-chlorofluorescein (HEX). PCR products were analyzed on an ABI3730 (Thermo Fisher Scientific, Waltham, MA, USA) automatic DNA sequencer using the software GENEMAPPER^®^, version 4.1.

### 2.3. Data Analysis

Data were analyzed for possible stuttering, drop out, and writing mistakes with the MICRO-CHECKER software version 2.2.3 [42]. Basic statistics for each population sample—the average number of alleles per locus (*N_A_*), allelic richness (*a*), fixation index or inbreeding coefficient (*F*_IS_), and unbiased heterozygosity estimates (*H*_E_, Nei’s gene diversity)—were computed with FSTAT version 2.9.3.2 [43]. Average frequency of null alleles (*Na*) was estimated using the FREENA program [44]. Deviations from Hardy–Weinberg equilibrium (HWE) at each locus and linkage disequilibrium after Bonferroni correction between each loci pairs were calculated with software GENEPOP version 4.2 [45].

Hierarchical partitions of the genetic variance (AMOVA) were performed using ARLEQUIN version 3.5.1.2 [46] from the 34 population samples arbitrarily grouped either according to their growing zones (central and southern Chile) and locations (26 locations) or according to their host plants (apple, pear, quince, and walnut) and locations. Differentiation between the Chilean central and southern zones was assumed, because central Chile corresponds to extensive industrial pome fruit production areas for export, while only traditional productions in small farms are found in southern Chile. In addition, apple, as the main host plant, was compared alone with the other three host plants in a group (pear, quince, and walnut). Significances of pairwise *F*_ST_ values were tested based on 1000 permutations of the multilocus genotypes.

Isolation by distance (IBD) was tested by regressing the Rousset’s genetic distances between the population samples (*F*_ST_/1 − *F*_ST_) and the logarithm of the geographic distances between the sample locations (Log km), and using Mantel’s test based on 1000 permutations of pairs of samples implemented in the XLSTAT software, version 7.5.2 [47].

Spatial genetic structure was inferred using Bayesian clustering method implemented in the TESS software, version 2.3 [48]. This method considers spatial coordinates of the genotyped individuals to assign them in relevant clusters. Models were computed for varying numbers of clusters (K_max_) from 2 to 20 assuming a convolution Gaussian prior for spatial admixture (BYM). For each model, 100 runs were computed with 10,000 sweeps, after a burn–in period of 5000 sweeps [48]. To estimate the number of clusters in the data, the highest likelihood runs were selected based on deviance information criterion (DIC) graphed against K_max_. Finally, the spatial distribution of clusters was plot using Voronoi Tessellation.

## 3. Results

### 3.1. Basic Statistics, HWE, and Linkage Disequilibrium

A total of 609 individuals from Chile and 109 from France were successfully amplified at the five microsatellite loci, which were all polymorphic in every population sample (Table 2). The number of alleles per locus ranged from three (*Cp5.24*) to 16 (*Cp.1.62*) for Chilean population samples and from three (*Cp5.24*) to 19 (*Cp6.46*) for the French population samples. The mean number of alleles per locus ranged from 3.0 to 5.2 over the 34 Chilean population samples, and from 6.2 to 9.0 for the three French population samples (Table 2). Allelic richness showed a similar result, with values ranging from 2.8 to 3.9 for Chilean population samples and from 4.3 to 4.7 for French population samples (Table 2). There was a low average proportion of null alleles in all population samples (Table 2).

The mean expected heterozygosity *H*_E_ ranged from 0.436 to 0.645, and the mean *F*_IS_ ranged from −0.215 and 0.140 over the 34 population samples from Chile (Table 2). For the three French populations, the *H*_E_ ranged from 0.664 to 0.696 and *F*_IS_ ranged from −0.060 and 0.117 (Table 2). Only three population samples from Chile (GulA, ColA, and PenA) and one from France (VleP) significantly departed from the Hardy–Weinberg equilibrium (*p* < 0.05). Significant linkage disequilibria were observed between *Cp1.60* and *Cp.2.129* (*p* < 0.001); *Cp1.62* and *Cp.2.129* (*p* < 0.001); *Cp1.62* and *Cp6.46* (*p* < 0.001); and *Cp5.24* and *Cp6.46* (*p* < 0.001).

### 3.2. AMOVA and F_ST_ Analysis

AMOVA detected a low but significant genetic differentiation between the central and southern Chilean zones (*F*_CT_ = 0.005, *p* ≤ 0.05), and between locations in each zone (*F*_SC_ = 0.025, *p* ≤ 0.001) that together accounted for by a 2.9% of the total genetic variance (Table 3). The main part of the genetic variance was distributed within each location (97.1%, Table 3). The hierarchical partition of the genetic variance was not significant between host plants, both when all four host-plant species were compared (*F*_CT_ = 0.0005, *p* > 0.05, not significant (N.S.)) (Table 3) and when apples were compared to the three other host-plant species together (*F*_CT_ = 0.0004, *p* > 0.05, N.S., table not shown).

**Figure 2:**
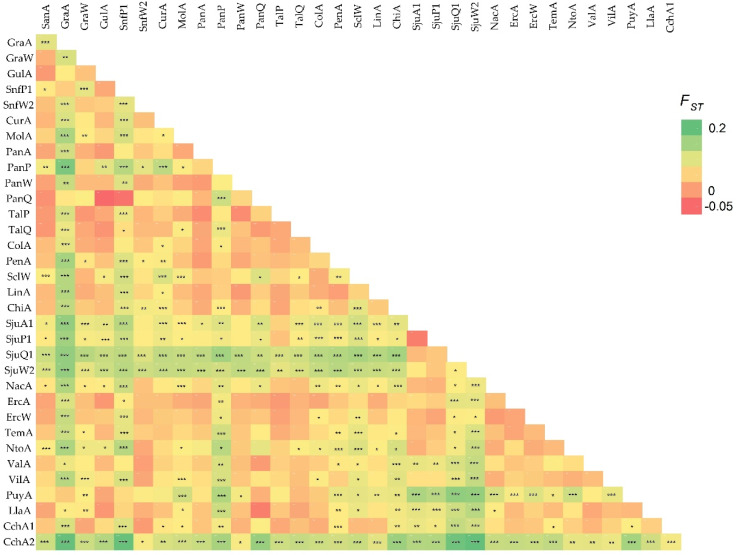
Pairwise genetic differentiation (*F*_ST_) values between codling moth samples between locations and host-plant species in Chile. Heatmap showing *F*_ST_ in different colors from red to green indicating lower or higher differentiation, respectively. Significant pairwise differentiation indicated with * = *p* ≤ 0.05, ** = *p* ≤ 0.01, and *** = *p* ≤ 0.001.

Pairwise comparison between the 34 Chilean population samples showed significant differentiation in 274 out of 561 combinations (48.8%) (Figure 2). Significant pairwise *F*_ST_ values ranged from 0.171 between CchA2 and SjuW2, to 0.015 between CurP and TalQ (Figure 2). The lowest *F*_ST_ negative value was −0.031 between PanQ and GulA (Figure 2). Eight population samples accumulated 69.0% of all the significant pairwise *F*_ST_ values. Among them, a group of four population samples located on the coastal range of the Bío-Bío Region showed significant differences that accounted for by 37.2% of the significant pairwise *F*_ST_ (SjuA1, SjuP1, SjuQ1, and SjuW2). When more than one host-plant species was present in the same locality, a significant differentiation only in three out of 14 possible pairwise comparisons between host plants within the same locality was found (Table 4). These samples were from GraA versus GraW (apple versus walnut), PanP versus PanQ (pear versus quince), and SjuW2 versus SjuQ1 (walnut versus quince) (Table 4).

**Figure 3:**
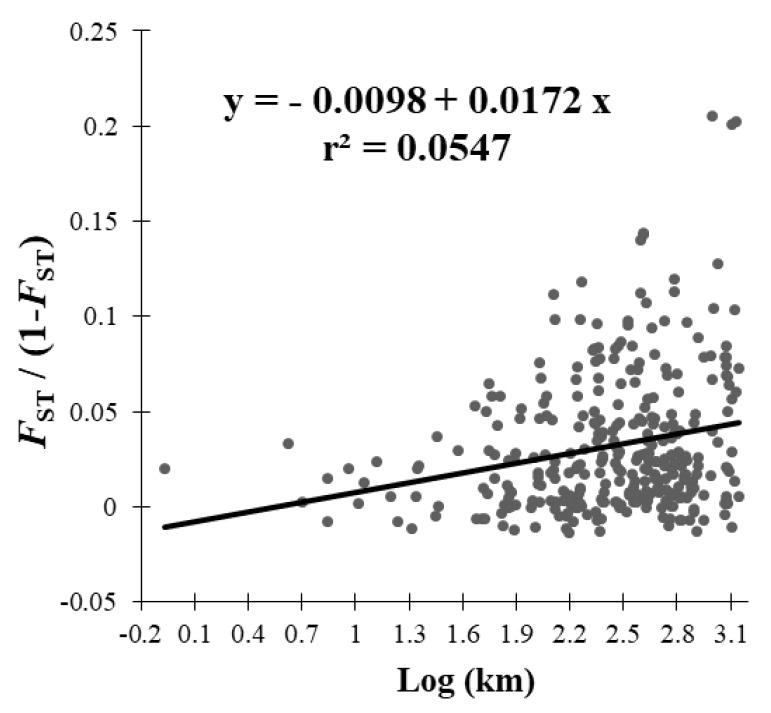
Geographic distance between sample localities versus linearized genetic differentiation between codling moth population samples, indicating significant isolation by distance.

The linear regression of the genetic distance and geographical distance between locations was positive and significant ((*F*_ST_/1 − *F*_ST_) = −0.0098 + 0.0172 Log km, r^2^ = 0.06, *p* < 0.001), in agreement with an isolation by distance of codling moth populations in Chile (Figure 3). The Mantel test revealed a significant positive correlation between genetic differentiation and geographic distance as well (r = 0.23, *p* < 0.001). Our southernmost locality CchA2 on the Aysén Region was significantly different from all remaining locations (Figure 2). Furthermore, CchA1, located very near to CchA2 (4.5 km), was significantly different from the latter, despite the fact that CchA1 had no significant differentiation between itself and many locations, including the most distant SanA location (1400 km north) (Figure 2).

### 3.3. Bayesian Cluster Analysis

**Figure 4:**
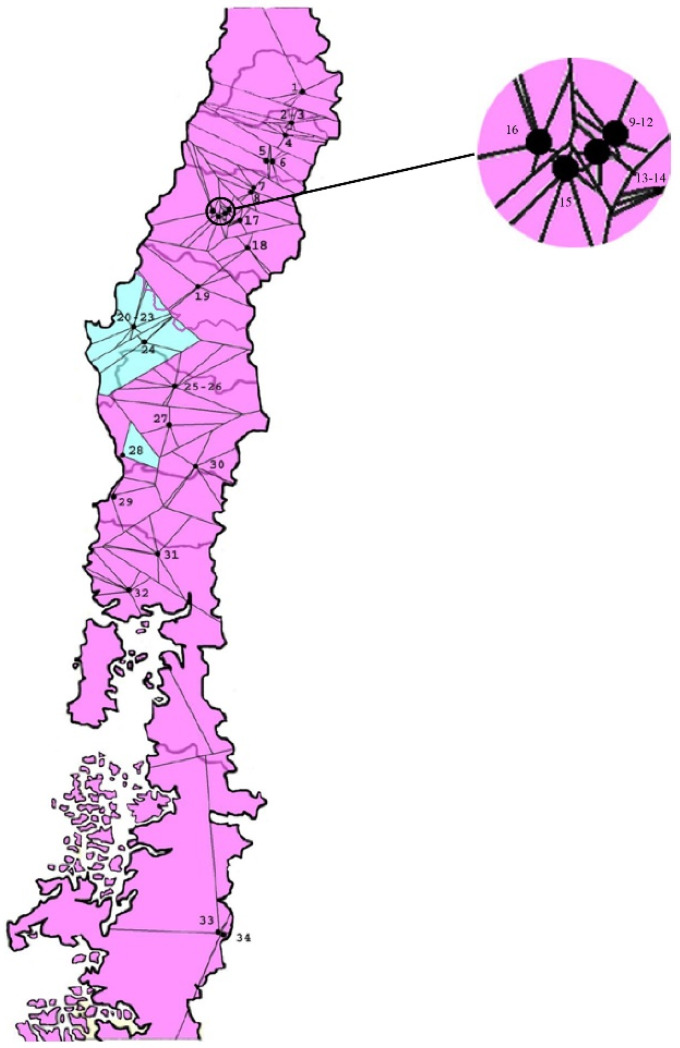
Voronoi tessellation of population structure in space of the codling moth, estimated using TESS. Number codes are detailed in Table 1. The map indicates groups of population samples with different colors. A group of population samples located at short distances is shown on a larger scale in the circle.

Our assignment test showed two clusters (K = 2) (Figure 4). The locations from the coastal range of Bío-Bío region (SjuA1, SjuP1 and SjuW2) were assigned in the same group, while all other locations were assigned into a large second group. This analysis was consistent with pairwise *F*_ST_, with the exception of the CchA2 sample, which was assigned to the large group.

## 4. Discussion

The present study included a wide latitudinal range (approximately 1400 km) that covered the main distribution of the codling moth in the west coast of South America [3], and the main host plant range of the codling moth in Chile [37,38]. We found significant but low levels of genetic differentiation between codling moth populations from different localities and regions. Interestingly, the genetic differentiation between localities found herein (*F*_ST_ = 0.03) was one to two orders of magnitude higher than in previous studies of codling moth in Chile (*F*_ST_ = 0.002 − 0.0001), covering a more restricted latitudinal range of approximately 180 km and using only apples as the host plant [14,31]. It is important to note that microsatellite markers used herein were different to those used in the previous studies from Chile. Significant linkage disequilibrium was found between four pairs of loci involving the five loci analyzed in our study. Previous research with locus *Cp1.62* has found significant linkage disequilibrium possibly related with selection of the *kdr* mutation, which confers resistance against pyrethroid insecticides [16]. Furthermore, a chromosome-level genome assembly for the codling moth has been recently published [49], from which the chromosomal positions of the microsatellite loci used in our study can be estimated. Based on this genomic information, we found that *Cp1.60* and *Cp1.62* are in chromosome 17 at positions 2,792,256 and 17,990,411, respectively. These microsatellite loci were not significantly linked, and therefore they are in rather distant positions on the same chromosome. The remaining three loci were found in different chromosomes (*Cp.2.129* chromosome 13, *Cp5.24* chromosome 24, and *Cp6.46* chromosome 5), and therefore, they are not physically linked. The significant linkage disequilibrium found in our study could be related with other genetic processes, such as drift or hitchhiking selection.

Based on pairwise *F*_ST_ analysis and hierarchical assignment, we detected a group of five population samples from three localities in the Bío-Bío Region, which were significantly different to all other codling moth populations from Chile. These population samples were composed by different host-plant species (apple, pear, quince, and walnut), suggesting that such a result is not associated with a host-plant-based genetic differentiation. Four of these population samples had less than 10 km between them in the Coastal Mountain Range of the Bío-Bío Region (latitude 37° S), in an isolated area from the main pome fruit production zone in the central valley of Chile.

Pome fruits in Chile were introduced by Spanish conquerors as early as the 16th century [39,50]. However, the first report of the codling moth in Chile dated from the last decade of the 19th century [38]. At this time, the southern part of Chile was in an active process of colonization by European immigrants mostly from Germany [51], and it was also a few decades before the area experienced a large trade relationship with California during the “gold rush” [52]. It is possible that the codling moth was introduced to Chile with pome fruit plant material brought either by European settlers, or from commercial trade with California where the codling moth was already present in 1872 [53]. A distinct codling moth population from the coastal range of Bío-Bío Region could have been isolated from other populations in the Chilean central valley since the beginning of the invasion process after over at least 300 generations. At present this coastal range is extensively covered by plantations of insigne pine (*Pinus radiata* D. Don) for the production of timber and paper [54], which might represent a barrier for the codling moth’s adult flight dispersal. These managed monoculture forest plantations do not present conditions for the growth of codling moth host plants; therefore, they could produce a barrier to dispersal similar to mountain areas described for Europe [10,23] or Iran [25] and desert areas for China [1,8,11,13].

A more recent codling moth invasion process occurred in the southernmost localities included in this study in the Aysén Region, where only during the last decade of the 20th century the introduction of this species was reported [36]. This southern region (latitude 46° S) has ocean influenced climate with cool temperatures and permanent rain, but close to the Andes Mountain Range, a small area near the lake General Carrera (Chile Chico) and close to the border with Argentina has a microclimate that allows fruit production for small farmers and local consumption [36]. Two localities less than 4.5 km away were sampled in this area, showing one of them to be similar (CchA1) and the other differentiated (CchA2) to the populations of the O’Higgins and Maule Regions in central Chile. This local genetic differentiation could suggest a multiple introduction process of the codling moth for one case from central Chile, and, in the other, a possibly different origin. The nearest fruit pome production area to Chile Chico is the Argentinean Patagonia with Neuquén and Rio Negro Regions, areas that could have been a source for codling moth based on the antique road connectivity of these Argentinean Patagonian Regions with Chile Chico that only in the last four decades was connected with the rest of Chile by land.

We did not find a significant genetic differentiation between samples from different host plants (pear, quince, and walnut). Previous studies using allozymes in France [55], microsatellites in Greece and southern France [30], and AFLP in South Africa [22], did not detect any differentiation in codling moth samples from different host-plant species. More recently, Chen and Dorn [10], using microsatellites, reported a significant genetic differentiation of an apricot codling moth strain in some regions of Switzerland. Similarly, Thaler et al. [23], using AFLP, found significant differentiation between apple and walnut samples from the same locality in Italy. In our study, comparisons between host-plant species in the same locality resulted in significant pairwise *F*_ST_ values only between apples and walnuts (Graneros), pears and quinces (Panguilemo), and walnuts and quinces (Santa Juana). However, another 11 possible comparisons of different host-plant species in the same locality did not present significant pairwise *F*_ST_ values (Figure 2). Furthermore, genetic differentiation was found between two population samples from apple from localities only 4.5 km away (CchA1 and CchA2) in the southernmost collection sites of Chile Chico (Figure 2). This difference could be explained by management practices (CchA1 unmanaged and CchA2 production for domestic market using insecticides) or different introductions, as discussed above.

Thus, overall genetic differentiation between codling moth from different host-plant species was not consistent between host-plant samples within localities. The potential introduction of codling moth populations from different host plants species should have resulted in a more consistent genetic differentiation related to the host plants between the different localities. Furthermore, body mass, wing size, and shape variables studies performed with geometric morphology techniques did not show significant differences between the body size or wing morphology of the codling moths obtained from different host-plant species (apple versus walnut), further supporting the lack of host-plant strains of this species in the Maule Region of Chile [56].

Finally, the codling moth is regarded as a sedentary pest with rather limited dispersal capacity in the adult stage, with the exception of a few genotypes with inherited long range flight behavior [57,58,59,60]. These attributes can produce a detectable pattern of genetic structure between different populations [1,8,10,13,22,23], but the low genetic structure detected in our study and others [4,15,16] is probably associated with long range passive dispersal associated with human cultivation of pome fruit worldwide.

## 5. Conclusions

Low genetic differentiation among the codling moth population samples was found, with only slight isolation by distance. According to a Bayesian assignment test (TESS), a group of localities in the Coastal Mountain Range from the Bío-Bío Region was found to conform to a distinct genetic cluster. We did not find significant genetic differentiation between codling moth samples from different host-plant species. Our results indicate high genetic exchange among codling moth populations between the different Chilean regions and host plants.

## Figures and Tables

**Table 1:** Code, location, region, and host plant of each codling moth sample genotyped from Chile and France.

	Code	Location	Region/Country Zone	Host	Latitude	Longitude
(1)	SanA	Santiago	Metropolitan C ^a^	Apple	33°31′57.5″ S	70°32′40.1″ W
(2)	GraA	Graneros	O’Higgins C	Apple	34°0.4′3.8″ S	70°42′43.7″ W
(3)	GraW	Graneros	O’Higgins C	Walnut	34°0.4′3.8″ S	70°42′43.7″ W
(4)	GulA	Gultro	O’Higgins C	Apple	34°11′51.9″ S	70°46′31.5″ W
(5)	SnfP1	San Fernando 1	O’Higgins C	Pear	34°36′8.5″ S	71°2′9.7″ W
(6)	SnfW2	San Fernando 2	O’Higgins C	Walnut	34°36′18″ S	70°58′43″ W
(7)	CurA	Curicó	Maule C	Apple	35°1′12.2″ S	71°14′26.2″ W
(8)	MolA	Molina	Maule C	Apple	35°5′52″ S	71°16′26.27″ W
(9)	PanA	Panguilemo	Maule C	Apple	35°22′13.4″ S	71°35′50.3″ W
(10)	PanP	Panguilemo	Maule C	Pear	35°22′13.4″ S	71°35′50.3″ W
(11)	PanW	Panguilemo	Maule C	Walnut	35°22′13.4″ S	71°35′50,3″ W
(12)	PanQ	Panguilemo	Maule C	Quince	35°22′13.4″ S	71°35′50,3″ W
(13)	TalP	Talca	Maule C	Pear	35°24′48.6″ S	71°38′24.2″ W
(14)	TalQ	Talca	Maule C	Quince	35°24′47.8″ S	71°38′24.1″ W
(15)	ColA	Colín	Maule C	Apple	35°27′56.5″ S	71°44′4.5″ W
(16)	PenA	Pencahue	Maule C	Apple	35°23′9.8″ S	71°48′38.4″ W
(17)	SclW	San Clemente	Maule C	Walnut	35°31′24.7″ S	71°26′0.3″ W
(18)	LinA	Linares	Maule C	Apple	35°57′10.7″ S	71°19′29.1″ W
(19)	ChiA	Chillán	Ñuble S	Apple	36°32′50.5″ S	72°1′27.6″ W
(20)	SjuA1	Santa Juana 1	Bío Bío S	Apple	37°10′11.1″ S	72°56′25.3″ W
(21)	SjuP1	Santa Juana 1	Bío Bío S	Pear	37°10′10.6″ S	72°56′25.4″ W
(22)	SjuQ1	Santa Juana 1	Bío Bío S	Quince	37°10′11.7″ S	72°56′25.9″ W
(23)	SjuW2	Santa Juana 2	Bío Bío S	Walnut	37°10′37″ S	72°56′13″ W
(24)	NacA	Nacimiento	Bío Bío S	Apple	37°24′21.1″ S	72°47′38.8″ W
(25)	ErcA	Ercilla	Araucanía S	Apple	38°5′29.5″ S	72°21′5.4″ W
(26)	ErcW	Ercilla	Araucanía S	Walnut	38°5′29.5″ S	72°21′5.4″ W
(27)	TemA	Temuco	Araucanía S	Apple	38°41′8.6″ S	72°25′37.5″ W
(28)	NtoA	Nueva Toltén	Araucanía S	Apple	39°9′35,7″ S	73°6′2.8″ W
(29)	ValA	Valdivia	Los Ríos S	Apple	39°46′29.7″ S	73°14′52.8″ W
(30)	VilA	Villarrica	Los Lagos S	Apple	39°78′86″ S	72°3′32″ W
(31)	PuyA	Puyehue	Los Lagos S	Apple	40°41′10″ S	72°35′45″ W
(32)	LlaA	Llanquihue	Los Lagos S	Apple	41°15′12″ S	73°0′12″ W
(33)	CchA1	Chile Chico 1	Aysén S	Apple	46°32′29.8″ S	71°43′21.7″ W
(34)	CchA2	Chile Chico 2	Aysén S	Apple	46°33′38″ S	71°40′25″ W
(35)	VleA	Valence	Avignon F	Apple	44°58′43″ N	4°55′45″ E
(36)	VleP	Valence	Avignon F	Pear	44°58′32″ N	4°55′53″ E
(37)	VleW	Valence	Avignon F	Walnut	44°58′31″ N	4°56′01″ E

^a^ C = central Chile, S = south Chile, F = France.

**Table 2:** Genetic variability at five microsatellite loci in the codling moth samples from Chile. Number of individuals per sample (*N*), mean number of alleles per locus (*N_A_*), allelic richness (*a*), average proportion of null alleles (*Na*), mean expected heterozygosity (*H*_E_), and mean inbreeding coefficient (*F*_IS_) for each sample.

Sample	*N*	*N_A_*	*a*	*N_a_*	*H*_E_	*F*_IS_
SanA	19	5.2	3.7	0.022	0.605	−0.184
GraA	20	3.6	2.8	0.000	0.496	−0.028
GraW	20	4.4	3.5	0.027	0.575	−0.147
GulA	17	4.2	3.5	0.002	0.608	−0.025 *
SnfP1	19	4.6	3.4	0.027	0.578	−0.049
SnfW2	20	4.4	3.5	0.006	0.565	0.140
CurA	19	4.8	3.5	0.000	0.565	0.020
MolA	19	5.0	3.8	0.027	0.644	−0.143
PanA	17	4.2	3.3	0.004	0.602	0.101
PanP	15	4.4	3.5	0.000	0.556	0.001
PanW	17	4.4	3.5	0.024	0.592	0.046
PanQ	7	3.0	3.0	0.006	0.588	−0.215
TalP	20	4.8	3.4	0.027	0.592	0.043
TalQ	19	5.2	3.6	0.018	0.568	−0.017
ColA	20	5.0	3.8	0.030	0.635	−0.087 *
PenA	20	4.2	3.3	0.009	0.582	−0.203 *
SclW	19	5.0	3.6	0.015	0.585	0.046
LinA	19	4.6	3.6	0.001	0.603	−0.082
ChiA	19	4.0	3.2	0.000	0.587	−0.087
SjuA1	15	3.8	3.2	0.009	0.558	−0.004
SjuP1	20	5.0	3.4	0.000	0.575	−0.061
SjuQ1	18	4.4	3.4	0.000	0.525	−0.078
SjuW2	19	4.4	3.2	0.006	0.535	−0.102
NacA	17	3.8	2.9	0.000	0.516	−0.048
ErcA	20	5.0	3.9	0.001	0.645	−0.117
ErcW	20	5.0	3.7	0.000	0.589	−0.031
TemA	17	4.2	3.3	0.006	0.583	−0.050
NtoA	8	3.8	3.7	0.032	0.566	−0.148
ValA	17	4.2	3.3	0.025	0.582	0.049
VilA	17	3.8	3.2	0.006	0.535	−0.078
PuyA	20	4.0	3.1	0.007	0.525	0.038
LlaA	19	4.8	3.6	0.000	0.591	0.056
CchA1	20	4.6	3.6	0.026	0.582	−0.095
CchA2	17	4.0	2.9	0.037	0.436	−0.149
VleA	29	7.6	4.7	0.000	0.696	−0.060
VleP	24	6.2	4.3	0.045	0.660	0.117 *
VleW	56	9.0	4.4	0.023	0.675	0.064

* Populations significantly departed from the Hardy–Weinberg equilibrium.

**Table 3:** Results of AMOVA of codling moth samples between locations and host-plant species in Chile.

Variation Source	df	Sum of Squares	Variance Components	Percentage of Variation	Fixation Index ^a,b^
Among groups (zone)	1	7.485	0.00652	0.44	*F*_CT_ = 0.00445 *
Among locations within groups (location)	24	74.320	0.03646	2.49	*F*_SC_ = 0.02499 ***
Within locations	1192	1695.813	1.42266	97.07	*F*_ST_ = 0.02932 ***
Total	1217	1777.618	1.46564	100	
Among groups (host plant)	3	9.252	0.00068	0.05	*F*_CT_ = 0.00046
Among locations within groups (location)	30	87.336	0.04172	2.85	*F*_SC_ = 0.02854 ***
Within locations	1184	1681.030	1.41979	97.10	*F*_ST_ 0.02899 ***
Total	1217	1777.618	1.46218	100	

^a^*F* index over all loci; ^b^ * indicates *p* ≤ 0.05; *** *p* ≤ 0.001.

**Table 4:** Pairwise genetic differentiation (*F*_ST_) values between codling moth samples between host-plant species in the same location in Chile. Comparisons among host-plant species from different localities are not shown.

	GraA	PanA	PanP	PanW	SjuA1	SjuP1	SjuQ1	ErcA
GraW	0.057 ^a^ **							
PanP		0.011	-					
PanW		−0.011	0.023	-				
PanQ		−0.001	0.068 **	0.012				
SjuP1					−0.023	-		
SjuQ1					0.001	0.006	-	
SjuW2					0.020	0.006	0.025 *	
ErcW								−0.008

^a^ * indicates *p* < 0.05, ** *p* < 0.01.
